# Genetic data improve the assessment of the conservation status based only on herbarium records of a Neotropical tree

**DOI:** 10.1038/s41598-019-41454-0

**Published:** 2019-04-05

**Authors:** André Carneiro Muniz, José Pires Lemos-Filho, Renata Santiago de Oliveira Buzatti, Priciane Cristina Correa Ribeiro, Fernando Moreira Fernandes, Maria Bernadete Lovato

**Affiliations:** 1 Departamento de Biologia Geral, Universidade Federal de Minas Gerais, CP 486, Belo Horizonte, MG 31270-901 Brazil; 2 Departamento de Botânica, Universidade Federal de Minas Gerais, Belo Horizonte, MG 31270-901 Brazil; 3 Jardim Botânico da Fundação Municipal de Parques e Zoobotânica de Belo Horizonte, Coordination of Plano de Ação Nacional para a Conservação do faveiro-de-wilson, Belo Horizonte, Brazil; 4 Present Address: Universidade Federal do Tocantins, Campus Universitário Araguaína, Araguaína, TO 77824-838 Brazil

## Abstract

Although there is a consensus among conservation biologists about the importance of genetic information, the assessment of extinction risk and conservation decision-making generally do not explicitly consider this type of data. Genetic data can be even more important in species where little other information is available. In this study, we investigated a poorly known legume tree, *Dimorphandra exaltata*, from the Brazilian Atlantic Forest, a hotspot for conservation. We coupled species distribution models and geospatial assessment based on herbarium records with population genetic analyses to evaluate its genetic status and extinction risk, and to suggest conservation measures. *Dimorphandra exaltata* shows low genetic diversity, inbreeding, and genetic evidence of decrease in population size, indicating that the species is genetically depleted. Geospatial assessment classified the species as Endangered. Species distribution models projected a decrease in range size in the near future (2050). The genetic status of the species suggests low adaptive potential, which compromises its chances of survival in the face of ongoing climatic change. Altogether, our coupled analyses show that the species is even more threatened than indicated by geospatial analyses alone. Thus, conservation measures that take into account genetic data and the impacts of climate change in the species should be implemented.

## Introduction

Habitat loss, overexploitation of natural resources, invasion by alien species, and global climate change are among the most important factors that threaten biodiversity, and can synergistically act to increase species extinction risk in the future^1–3^. Information used to decide if a species is at risk of extinction, and what threatened category it falls into is generally based on ecological and demographic data such as the number of known individuals, actual or projected declines in population size, and the extent of occurrence (EOO) or area of occupancy (AOO)^4^. The EOO is an estimate of the total area currently occupied by a taxon and the risks within this area^4^. The AOO is a metric of current occupation of suitable habitats within the EOO, and measures the risks to a taxon occurring within small patches or in few patches within the EOO^4^. Demographic information is fundamental in conservation biology, but in several species, it is difficult to directly estimate.

There is a consensus among conservation biologists as to the importance of genetic factors in determining the fates of populations or species, but these factors are not generally used to define the conservation status of a species^5,6^. The “genetic health” of a population or species is dependent on its genetic diversity and inbreeding level. Rare and endangered species tend to have reduced genetic diversity within populations and low gene flow rates among them, because in general the populations are small and highly disjointed^7^. Furthermore, habitat loss and fragmentation processes can lead to the disruption of pollination and dispersion, resulting in low reproductive success and making populations more isolated and prone to inbreeding depression, i.e., a reduction in the capacity for survival and reproduction of the offspring of inbreeders^5,8^.

The genetic diversity and degree of inbreeding in a population are directly dependent on the effective population size (N_E_), a fundamental parameter for the conservation of threatened species^9–11^. The N_E_ of an actual population can be defined as the size of an ideal population that suffers the same magnitude of genetic drift as the actual population; this genetic drift is generally measured as a loss of heterozygosity, increase of identity by descent, or change in allele frequency over time^12^. The difficulty and impracticality of directly counting individuals in most species, coupled with the importance of genetic parameters in putatively threatened species, led to the use of N_E_ in place of census population size in species conservation. Furthermore, several population genetic tools based on coalescent theory are currently available for estimating the temporal dynamics of N_E_ and detecting population genetic structure and migration rates among populations^13–15^. Information about these genetic parameters could be used to gain knowledge about how global habitat loss, fragmentation and novel environment will affect a species and thus help to optimize its conservation^16–20^. These genetic studies are even more important in poorly known species, as they could help to infer a species’ conservation status and aid in making decisions about its conservation.

**Figure 1:**
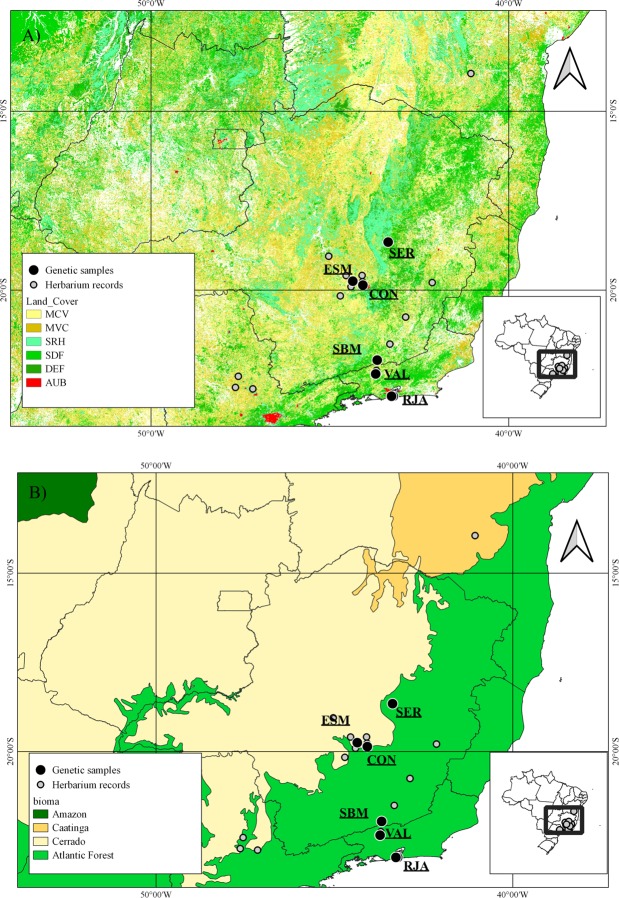
Maps showing the *Dimorphandra exaltata* herbarium records and the populations used for genetic analyses. (**A**) Map showing the land use of eastern Brazil (ESA/CCI viewer, 2018), MCV = mosaic cropland (50–70%)/vegetation (20–50%), MVC = mosaic vegetation (50–70%)/cropland (20–50%), SDF = evergreen or semi-deciduous forest (>5 m), DEF = deciduous forest (>5 m), SRH = herbaceous vegetation, AUB = Urban areas > 50%. (**B**) Map showing the Brazilian biomes where *Dimorphandra exaltata* occurs.

In this study, we investigated the poorly known legume tree *Dimorphandra exaltata* Schott (Fabaceae, Caesalpinioideae). Although *D. exaltata* is the type species of *Dimorphandra* as described by Schott in 1827^21^, there are only a few herbarium records (Fig. 1) and almost no studies have been performed with it^22^. The species has a discontinuous distribution in semi-deciduous forests in the Atlantic Forest biome and Atlantic Forest/Cerrado transition areas in southeastern Brazil (Fig. 1). Atlantic Forest and Cerrado (a savanna biome) are hotspots for biodiversity conservation due to the high degree of threats, species richness, and endemism, with each biome showing about 8 000 and 4 400 endemic plant species, respectively^23^. After five centuries of human exploitation since the Portuguese colonization of Brazil, only 12–15% of the Atlantic Forest remains, in small and isolated fragments^24^. Deforestation and fragmentation in Cerrado is more recent, but has occurred in high rates; from 1970 to 2005 about 50% of the biome land cover was transformed into pasture and agricultural land^25^. *Dimorphandra exaltata* has a distribution partially sympatric with that of the critically endangered species *D. wilsonii*^26^. These areas have been highly disturbed by anthropogenic activities, mainly agriculture and urbanization^27^.

Here, we performed population genetic analyses to assess the genetic status of *D. exaltata*, using nuclear microsatellite markers. We have genotyped a total of 62 individuals from: two populations in transition areas of Cerrado/Atlantic Forest, one population from semi-deciduous Atlantic Forest and seven isolated individuals from three sites in eastern Atlantic Forest (Fig. 1; Table 1). We estimated the genetic diversity and evaluated genetic structure with Bayesian clustering method, as well as the genetic connectivity among extant populations and historical N_E_ using coalescent methods. We looked for genetic evidence of decreases in population size and evaluated the contemporary N_E_ through of molecular coancestry. Further, we compared the genetic status of *D. exaltata* with its conservation status evaluated using geospatial assessment based on herbarium records, with estimates of EOO and AOO. We also estimated its current and near-future (2050) potential geographical distribution to investigate current habitat suitability and project the impact of climate change on the species. Based on the genetic and ecological data, we suggest conservation measures for the species.

**Table 1:** Population genetic diversity parameters of *Dimorphandra exaltata*.

Population	Code	Latitude	Longitude	*N*	*A*	*A*_*R*_	*H*_*O*_	*H*_*E*_	*F*_*IS*_	*A*_*P*_
Serro	SER	18 38′ S	43 22′ W	20	4.364	2.731	0.328	0.406	**0.195**	16
Contagem	CON	19 51′ S	44 04′ W	22	4.545	2.746	0.360	0.396	**0.178**	20
Esmeraldas	ESM	19 45′ S	44 21′ W	13	3.091	2.348	0.320	0.289	0.098	4
Isolated individuals in Atlantic Forest	AFI	22 57′ S	43 16′ W	7	3.800	3.419	0.481	0.560	—	9
		22 20′ S	43 43′ W							
		21 57′ S	43 40′ W							
Mean	—	—	—	18	3.886	3.205	0.372	0.413	0.157	12.25

*N* = number of individuals, *A* = mean number of alleles, *H*_*O*_ = observed heterozygosity, *H*_*E*_ = expected heterozygosity, *A*_*R*_ = allelic richness, *F*_*IS*_ = inbreeding coefficient, *A*_*P*_ = number of private alleles. *F*_*IS*_ values significantly different of zero (P < 0.05) are in bold.

## Results

### Genetic status: genetic diversity and structure, migration, and effective population size

Of the 11 microsatellites loci used, only Dw103 for CON population and Dw28 for all populations showed null alleles, while scoring errors due to stuttering or large allele dropout were not found. However, as the genetic diversity estimates were similar between the original dataset and the corrected for null alleles dataset using the Brookfield 1 method in MicroChecker version 2.2.0.2^28^, we considered the estimates without null allele correction. No significant linkage disequilibrium between loci was observed in any comparison after Bonferroni correction. To estimate the genetic diversity and structure of *D. exaltata* we also included the seven isolated individuals of the Atlantic Forest in addition to the three sampled populations. These individuals from three sites with distances ranging from 31.5 to 95.7 km were analysed together (AFI group; Table 1) to represent the diversity of eastern Atlantic Forest, although they probably do not represent samples of only one population. For the remaining genetic analyses, the AFI group was not included.

Among the three populations and the AFI group, the mean *A* and *A*_*R*_ ranged from 3.091 to 4.545 and from 2.348 to 3.419, respectively. *H*_*O*_ and *H*_*E*_ per population ranged from 0.320 to 0.481 and from 0.289 to 0.560, respectively, with mean values of *H*_*O*_ and *H*_*E*_ of 0.372 and 0.413, respectively (Table 1). Populations CON and SER showed the higher values of private alleles with 20 and 16 alleles, respectively. In addition, nine private alleles were found in the isolated individuals from Atlantic Forest, despite the small sample size (Table 1). The exact test showed that two of the three populations, SER and CON, showed significant positives inbreeding coefficients (*F*_*IS*_) after Bonferroni correction, with estimated values of 0.195 and 0.178 (P < 0.05), indicating deviation from Hardy-Weinberg equilibrium in these populations (Table 1).

**Figure 2:**
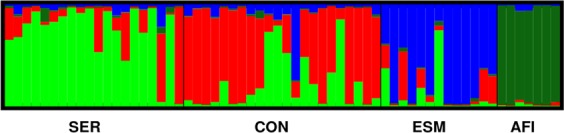
Bar plot showing the membership coefficients according to the Bayesian clustering method (STRUCTURE) for individuals of three *Dimorphandra exaltata* populations and seven isolated individuals (AFI) based on the best number of K = 4 genetic clusters.

AMOVA estimated *F*_*ST*_ of 0.180 (P = 0.000) and *R*_*ST*_ of 0.120 (P = 0.000), indicating moderate genetic divergence among populations (Table 2). According to pairwise comparisons, AFI followed by ESM population were the most divergent (Supplementary Table 1). The best number of clusters estimated by Bayesian clustering (Structure), considering both ΔK and mean likelihood methods, was K = 4 with each population been represented mostly by one genetic ancestry and varying admixture levels among them (Supplementary Fig. 1; Fig. 2). The CON followed by SER population showed higher admixture levels in its individuals (68% and 65% of individuals with Q < 0.9 for its most representative cluster, respectively), and AFI the lowest levels of admixture in the relation to other populations (Fig. 2). In addition, the Bayesian clustering showed that SER and CON were more related with each other, followed by ESM. AFI was the most divergent from other populations. These relationships are identified with K = 4 groups, but also when is considered grouping using K = 3, K = 5 and K = 6 genetic ancestries (Supplementary Fig. 2).

**Table 2:** Analysis of molecular variance of *Dimorphandra exaltata* based on *F*_*ST*_ and *R*_*ST*_. Significant values (P < 0.05) are in bold.

Source of Variation	d.f	Variance components (*F*_*ST*_)	% of variation (*F*_*ST*_)	Variance components (*R*_*ST*_)	% of variation (*R*_*ST*_)
Among populations	3	0.46	17.95	17.7	12.03
Within populations	120	2.09	82.05	129.46	87.97
Total	123	2.55		147.16	
Fixation Index			*F*_*ST*_ = **0.180**		*R*_*ST*_ = **0.120**

The contemporary N_E_ estimated for the three populations (SER, CON, ESM) ranged from 13.5 to 22.7, indicating low number of reproductive individuals (Table 3). The bottleneck test showed significant deviation of mutation-drift equilibrium in the three mutation models tested for all three populations (P < 0.05; Table 3), indicating that they suffered recent reductions in population size. The best historical migration model among the three populations was the full matrix migration (Supplementary Table 2). The values of the effective number of migrants (N_E_*m*) ranged from 0.086 from SER to ESM to 5.653 from ESM to SER (Supplementary Table 3). The estimated historical N_E_were 481, 760, and 11 for SER, CON, and ESM, respectively (Supplementary Table 3).

**Table 3:** Contemporary effective population sizes (N_E_) and results of tests for recent bottlenecks in *Dimorphandra exaltata* populations.

Population	N_E_	IAM	TPM	SMM	N	k
SER	22.7	**0.007**	**0.009**	**0.009**	39.82	2.55
CON	22.1	**0.005**	**0.005**	**0.016**	42.91	2.55
ESM	13.5	**0.002**	**0.007**	**0.010**	25.45	2.36

Values are the probability of a Wilcoxon-sign rank test of comparison between population heterozygosity under Hardy-Weinberg equilibrium and that under mutation-drift equilibrium. IAM = infinite allele model, TPM = two phase model, SMM = stepwise mutation model, N = mean number of gene copies per sample, k = mean number of alleles under mutation-drift equilibrium. Significant values (P < 0.05) are in bold.

### Species distribution models

**Figure 3:**
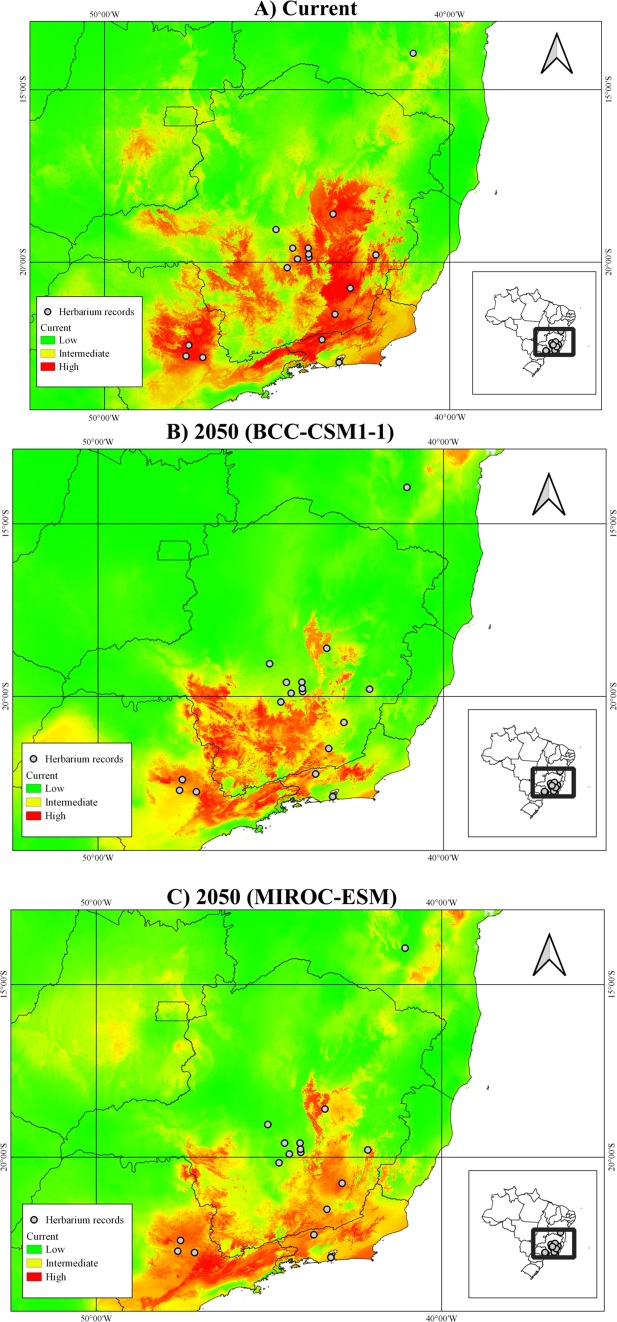
Ensemble species distribution model showing projections of suitable areas for *Dimorphandra exaltata*: (**A**) in the current time and projected into the near future (2050) based on the (**B**) BCC-CSM1-1 and (**C**) MIROC-ESM general circulation models of climate change.

Of the 19 bioclimatic variables from the WorldClim^29^, the following variables were retained to performing species distribution models after the factor analyses: mean diurnal range (Bio2), isothermality (Bio3), mean temperature of warmest quarter (Bio10), precipitation of wettest quarter (Bio16), and precipitation of driest quarter (Bio17). The values of TSS and AUC for model evaluation ranged from 0.333 to 1.000 and 0.611 to 1.000, respectively (Supplementary Tables 4 and 5). The variables that most contributed to the models were Bio3, Bio10, and Bio17 (Supplementary Table 6). The SDM projections confirmed the pattern of occurrence for *D. exaltata*, showing higher suitability in the semi-deciduous Atlantic Forest and in the Atlantic Forest/Cerrado transitional zone in southeastern Brazil, mainly in Minas Gerais, with smaller areas in São Paulo and Rio de Janeiro states (Fig. 3). The projections of both models for 2050 showed a loss in suitable area, mainly in the northern and northeastern parts of the range, which was more accentuated in the less-conservative BCC-CSM1-1 model (Fig. 3). In the most conservative scenario of climate change, MIROC-ESM, we observed a displacement of suitability toward the south, the opposite to a decrease in suitability in the southern area in the less-conservative model. There was also a decrease in suitability in the Atlantic Forest/Cerrado transitional zone, the area of most of the registered occurrences of the species (Fig. 3).

### Geospatial conservation assessment

Using the Geospatial Conservation Assessment Tool software^30^ (GeoCAT) based on 19 herbarium records to evaluating the conservation status of *D. exaltata*, we estimated an extent of occurrence (EOO) of 263 905 km^2^ and a much smaller area of occupancy (AOO) of 76 km^2^ for the species. Due to the possibility of misidentification in the only record for *D. exaltata* in *speciesLink* at Jequié, Bahia state, and because we could not find any other description for the species in Bahia in specialized literature^21,31,32^, we also performed the EOO and AOO analyses without this record. The estimates of EOO and AOO without Jequié were 174 936 km^2^ and 72 km^2^, respectively, similar to estimates with the complete dataset. Based on the B criteria of the IUCN, the species was classified as Low Concern according to the EOO (EOO > 20 000 km^2^) and as Endangered according to the AOO (AOO < 500 km^2^) in both analyses, with and without Jequié. In addition, following the B2 criteria, the species should also be considered Endangered because it meets the two following conditions: (a) severely fragmented populations and (b) continuous decline in area as indicated by (bi) projected EOO and (biii) continuing decline in area, extent, and/or quality of habitat.

## Discussion

Our study showed that *D. exaltata* exhibits low genetic diversity, inbreeding, and evidence of a recent decrease in population size. Furthermore, species distribution models project a reduction in range size for the near future (2050). According to the geospatial criteria of the IUCN (2014), the species falls into the Endangered category of extinction risk. Altogether, our data indicate clearly that *D. exaltata* is a threatened species of the Atlantic Forest. The contribution of genetic data, in addition to ecological data, to establishing the extinction risk of the species and the importance of obtaining both types of data to establish measures of conservation are discussed below.

*Dimorphandra exaltata* populations have lower genetic diversity (*H*_*E*_ = 0.289–0.560) than populations of several other legume tree species from the Atlantic Forest, including *Hymenaea courbaril* (*H*_*E*_ = 0.536–0.804)^33^, *Centrolobium tomentosum* (*H*_*E*_ = 0.500–0.550)^34^, *Inga vera* (*H*_*E*_ = 0.655–0.865)^35^, *Copaifera langsdorffii* (*H*_*E*_ = 0.881–0.893)^36–38^, and the vulnerable *Dalbergia nigra* (*H*_*E*_ = 0.682–0.798)^39–41^, all species evaluated with microsatellite markers. Another appreciation of the level of genetic diversity of *D. exaltata* could be based on a comparison with closely related species, which discounts the phylogenetic effect^42,43^. *Dimorphandra exaltata* populations exhibited considerably lower genetic diversity (mean *H*_*E*_ per population of 0.413) than a widespread congeneric species, *D. mollis*, which shows a mean *H*_*E*_ per population of 0.594^44^, as expected due to its more restricted distribution. *Dimorphandra exaltata* populations showed even lower diversity than the narrowly endemic and critically endangered congeneric species *D. wilsonii*, with a mean *H*_*E*_ per population of 0.559^27^. Although our genetic diversity evaluation was limited to 62 individuals, from three populations and isolated individuals from Atlantic Forest due to the rarity of the species, the low genetic diversity found indicates that *D. exaltata* is genetically depleted.

The contemporary N_E_ of *D. exaltata* populations ranged from 13.5 to 22.7 and the estimated historical N_E_ ranged from 11 to 700, which points to a low N_E_ for the species. The comparison between the two N_E_ together with the bottleneck tests point to a severe decrease in the population sizes of the species, what is probably associated with habitat loss and fragmentation in the study area. This pattern can explain the low diversity found in *D. exaltata*, since N_E_ is one of the most relevant parameters to explain the patterns of genetic variation in wild populations^11^. These results are very important for the management of endangered species since N_E_ of 100 and 1 000 individuals are suggested to prevent loss of genetic diversity by genetic drift for five generations and to maintain long-term evolutionary potential, respectively^45^.

The population genetic structure of *D. exaltata* suggests considerable admixture among populations, except for the isolated individuals of the Atlantic Forest. The historical demographic analysis reinforces that the three populations were genetically connected in the past. However, we should take care in extrapolating this connectivity to the current time. In fact, during our fieldwork, we observed a very low number of individuals and they were isolated and separated by large spatial distances, mainly in eastern Atlantic Forest. Also, the land use maps showed great anthropic pressure in the areas of occurrence of the species^46^ (Fig. 1), which results in fragmented habitats and isolated populations. As consequence of this fragmentation, the gene flow of *D. exaltata* is likely compromised by a low-quality matrix among fragments. The seed dispersion of the species can be affected by a shortage of dispersers. Two species of the genus, *D. mollis* and *D. wilsonii*, exhibit low seed dispersal, which is mainly performed by tapirs (*Tapirus terrestris*), which are vulnerable and locally extinct in some areas^26^. Although the seed disperser of *D. exaltata* is not known, the similarity among species of the genus suggests a similar pattern for it. This is a matter of concern because at least 1.0 migrant per generation is necessary to counteract the genetic drift effects in the loss of diversity of populations^47^.

Our populations showed significant inbreeding coefficients, indicating a departure from random mating within populations. Although the mating system of *D. exaltata* is not known, it is probably similar to that of the congeneric *D. wilsonii*, which has self-compatibility^26^ but mainly performs outcrossing^48^. The inbreeding found in *D. exaltata* may be due to selfing or mating between related individuals (biparental inbreeding). The small population size could favor mating among relatives or selfing in opposition to outcrossing. Increased selfing in preferentially outcrossing species is commonly found after fragmentation^5^. Furthermore, perennial plants, that have recently undergone habitat loss and fragmentation, such as *D. exaltata*, have a high probability of suffering inbreeding depression^8^. Therefore, inbreeding might be an additional threat to *D. exaltata*.

It is well-established that species with isolated and small populations are subject to loss of genetic diversity, a higher degree of inbreeding, and an elevated extinction risk in the wild^45^. Thus, the genetic data gathered here for *D. exaltata*, which showed low genetic diversity, inbreeding, and a recent reduction in population size, indicate that the “genetic health” of the species is compromised, which may decrease its adaptive capacity and threaten its survival in the wild in the near future. Thus, the genetic analyses of the species itself allow us to classify *D. exaltata* as a threatened species. In addition, the genetic data suggest that the species is more threatened than indicated by geospatial analyses.

The projected distribution for *D. exaltata* in the near future (2050) suggests a decrease in the total area of occurrence, including in the area in which it is most found currently, along with a displacement toward the south. The climate is considered to be the main driver of the species’ range extent at large scales and of its long-term dynamics^44,49–53^. In face of climate change, the probability of survival of a species is dependent on its capacity to adapt to the new environmental conditions or to migrate to suitable habitats^54^. However, the genetic data showed that the adaptive potential of *D. exaltata* is probably compromised. Thus, the species may depend on its migration capacity to colonize new favorable areas to survive in the face of climate change. However, the current connectivity among populations of the species is likely compromised by both low seed dispersal and pollen flow. Thus, the projected reduction of areas of occurrence and their displacement may be a concern for *D. exaltata*.

The geospatial assessment analyses showed that *D. exaltata* should be classified as Endangered, according the B2 criteria^4^ (AOO less than 500 km^2^). Furthermore, it could be also considered to be in the Threatened category because the populations are severely fragmented and there is a projected decline of this area, along with the low quality of its habitat (the sub criteria of B2). The Atlantic Forest has a fragmented distribution, with a history of disturbance and low functional connectivity among fragments^24^. Furthermore, the *D. exaltata* areas of occurrence in the Atlantic Forest/Cerrado transition zone, where the species is partially sympatric with *D. wilsonii*, are dominated by anthropogenic landscapes due to conversion of forested areas to agricultural lands, urbanization, and fire. These are the main threat factors for *D. wilsonii*^27^ and may also play this role for *D. exaltata*.

Although *D. exaltata* occurs in one of the most-inventoried regions of southeastern Brazil, only 19 occurrence records were found, demonstrating the rarity of the species. In several areas of Atlantic Forest/Cerrado transition, *D. exaltata* occurs together with *D. wilsonii*, a critically endangered species, which has a National Action Plan for its conservation^27^ (PAN faveiro de Wilson). It is highly recommended that this Action Plan also include *D. exaltata*, as the two species share similar ecological requirements and the same threat factors. Furthermore, only three records for *D. exaltata* were within protected areas. Protecting areas where the two *Dimorphandra* species co-occur will lead to protection of both threatened trees.

The genetic analyses indicated that the main risk factors to wild persistence of *D. exaltata* are its low genetic diversity and its low effective population size. Considering the ecological and genetic risk factors, conservation actions are urgently needed to decrease the probability of species extinction. Firstly, we recommend more search to find new individuals/populations of the species, mainly in easternmost areas Atlantic Forest. In addition, we recommend reintroduction, *ex situ* conservation, and genetic rescue to increase genetic diversity and maintain genetic connectivity among its populations. Genetic rescue is a highly beneficial strategy for management of genetically depleted populations^55^. For this, the creation and permanent maintenance of *ex situ* production of saplings from seeds of trees from different populations is a priority. As the analysed populations are not very genetically divergent, saplings from different populations could be introduced into the same locality, which could increase the genetic diversity and decrease the rate of inbreeding. However, considering the high genetic divergence of individuals from the eastern Atlantic Forest in relation to other areas, we suggested reintroduction in the eastern Atlantic Forest preferentially with saplings produced by seeds from these locations. The saplings should be used to increase the sizes of current populations, which must be protected, and to reintroduce the species into suitable areas according to SDM projections. It should be noted that the current suitable areas will suffer significant reduction in even the most optimistic projection of climate changes. Thus, it is recommended to take in account this projection when choosing localities for reintroduction.

## Conclusion

Our integrative genetic and ecological framework to assess the degree to which a poorly known tree species is threatened and to guide conservation measures for that tree species, which occurs in the Atlantic Forest, a large and highly threatened biome in eastern South America, was demonstrated to be highly effective. This strategy of investigation, which includes genetic data, could be used for species in other conservation hotspots worldwide, where generally there is a lack of demographic information to establish the conservation status of species. From the results, our integrative framework supplies essential information for conservation policies.

## Materials and Methods

### Study species

*Dimorphandra exaltata* is a medium to tall tree (15–30 meters), with bipinnate leaves, opposite leaflets pairs, and densely hairy petioles. Its inflorescences have a corymbe-type panicle consisting of multiple spikes. The flowers are cream or yellow with a glabrous chalice. Its fruits are flat legumes, which are almost straight, woody, and dark, indehiscent with sub-cylindrical seeds^21^. It flowers during November–December and fruit maturation occurs in September–October^21^. The pollinator and seed disperser of *D. exaltata* are not known. However, it has been proposed^21^ that the pollination is performed by insects because of the similarity of its inflorescences and flowers with those of the congeneric species *D. vernicosa*, which is pollinated by bees of the Apidae family.

### Herbarium records and study area

Our study initially evaluated the occurrence of *D. exaltata* through analyses of herbarium records. Georeferenced herbarium records of *D. exaltata* were obtained mainly from the *speciesLink* database^56^, but to check for duplicates and to add missing records, other databases were used as well^21,31,32^. Due to the similarities among several species of the genus, the records of *D. exaltata* were compared with those of other *Dimorphandra* species to avoid misidentification. We retained only the most recent records by locality to avoid bias due to differential sampling efforts in certain areas and aggregated surveys. The localities were defined as the exact coordinates of the sites available in the record or alternatively, when the coordinate of the site was not available, by the municipalities coordinates. After the filtering process, we obtained only 19 georeferenced records in the *speciesLink* database (Supplementary Table 7 and Fig. 1), mostly in the semi-deciduous Atlantic Forest and in the Atlantic Forest/Cerrado transition zone. In the transition zone, the occurrences were mainly in cropland or urbanized areas^46^ (Fig. 1). In addition, most of the peripheral records at the southern and eastern edges of occurrence were from near highly urbanized areas^46^ (Fig. 1). For species distribution models (SDM) and geospatial conservation assessment analyses, we used the 19 records.

We also made a great effort to collect *D. exaltata* on Minas Gerais and Rio de Janeiro states, which concentrate the highest number of herbarium records and the most potential distribution areas for the species (Figs 1 and 3). In our collect expeditions in the eastern of Minas Gerais and the Rio de Janeiro, we found only four juveniles in one area and three isolated adults in two distant sites with distances ranging from 31.5 to 95.7 km apart from each other evidencing that the species is rare.

For the genetic analyses, we sampled 55 individuals of *D. exaltata* from three populations and seven isolated individuals from the three sites in eastern Minas Gerais and Rio de Janeiro, totaling 62 individuals (Fig. 1, Table 1). Two populations are located in Atlantic Forest/Cerrado transition areas, Contagem (CON) and Esmeraldas (ESM), where the species occurs in higher density and the third population is located in Serro (SER) in the semi-deciduous Atlantic Forest (Fig. 1). The isolated individuals of the species were in two sites already recorded in the *speciesLink* database for the species, Rio de Janeiro (RJA) and Valença (VAL) and in a municipality that do not had register for *D. exaltata* occurence, Santa Bárbara do Monte Verde (SBM) in the semi-deciduous Atlantic Forest. These areas are highly fragmented and disturbed due to urban pressure and soil conversion to croplands^46^ (Fig. 1). The seven isolated individuals from RJA, VAL and SBM were used as representatives of the eastern Atlantic Forest, and were analysed together (AFI group), although they probably do not represent samples of only one population. AFI group was used only for estimates of genetic diversity and evaluation of genetic structure of species through of the analysis of molecular variance (AMOVA) and by the Bayesian clustering method implemented in STRUCTURE software^57,58^. The further genetic investigation was performed using only the populations SER, CON and ESM.

### DNA isolation and microsatellite genotyping

Frozen leaf or vascular tissue was used for DNA extraction according to the cetyltrimethylammonium bromide (CTAB) protocol^59^, slightly modified as suggested by^60^. Individuals were genotyped for 11 microsatellite loci, five isolated in *D. mollis*^61^ (Dmo5, Dmo7, Dmo13, Dmo20, Dmo21) and six isolated in *D. wilsonii*^62^ (Dw21, Dw28, Dw33, Dw105, Dw52 and Dw103) Amplifications of all markers were performed according to^61^. The amplicons were submitted to capillary electrophoresis in an ABI prism 3500xl automated sequencer with GeneScan TM ROX500TM as a standard fragment (Applied Biosystems, Foster City, CA, USA) and genotyped in GeneMapper software Version 5.0 (Applied Biosystems). Scoring errors due to the presence of null alleles, stuttering, or large allele dropout were evaluated in MicroChecker version 2.2.0.2^28^. Linkage disequilibrium for each pair of loci for each population was tested using a randomization-based test in FSTAT version 2.9.3.2^63^.

### Genetic status: genetic diversity and structure, migration, and effective population size

The genetic diversity of *D. exaltata* populations was estimated by the following parameters: mean number of alleles per locus (*A*), expected and observed heterozygosity (*H*_*E*_ and *H*_*O*_ respectively) using ARLEQUIN 3.5^64^ and the allelic richness (*A*_*R*_) using the rarefaction method of^65^ in FSTAT version 2.9.3.2^63^. The number of private alleles were investigated in GenALEx 6.503^66,67^. The inbreeding coefficient (*F*_*IS*_) was calculated and departures from Hardy-Weinberg expectations were tested with exact tests in FSTAT version 2.9.3.2^63^, with significance assessed after sequential Bonferroni correction for multiple comparisons. The *F*_*IS*_ was not estimated for the AFI group because they can represent samples of more than one population. Analyses of molecular variance (AMOVA) implemented in ARLEQUIN 3.5^64^ were used to estimate the hierarchical genetic structure and the pairwise *F*_*ST*_ and *R*_*ST*_ among populations.

The number of genetic clusters was evaluated using a Bayesian clustering method implemented in STRUCTURE version 2.3.4^57,58^. Parameter sets assumed an admixture model with correlated allele frequencies among patches. Ten independent simulations were run with 100 000 chains as burn-in and 1 000 000 Markov chain Monte Carlo (MCMC) replicates after burn-in, testing for K = 1–8. To evaluate the best K number, the mean log likelihood of the model through all runs and ΔK statistics were used^68^. To visualize the individual ancestry proportion, the average of ten runs for K was calculated in CLUMPP version 1.1.2^69^ and the bar plot membership coefficient of each individual was built in DISTRUCT 1.1^70^.

We estimated the contemporary effective populations sizes (*N*_*E*_) using the relation *N*_*E*_ = 0.5/Θ^71^; Θ is the group molecular coancestry for the total number of sampled trees in each population^72^. The calculation of Θ was made using the equation (1), $\Theta =\frac{{\sum }_{i=1}^{n}0.5(1+Fi)+{\sum }_{i=1}^{n}{\sum }_{j\ne i}^{n}\theta ij}{{n}^{2}}$^72^; *F*_*i*_ is the inbreeding coefficient of the *i-th* individual, *θ*_*ij*_ is the pairwise kinship between the individual *i-th* and *j-th*^38^ and *n* is the sample size. *F*_*i*_ and *θ*_*ij*_ were estimated in SPAGeDi 1.1 software^73^. We evaluated for recent reductions in population size using a test for heterozygosity excess implemented in BOTTLENECK version 1.2.02^74^. Populations that have suffered a recent size decrease exhibit reductions of allele numbers and heterozygosity. However, reduction in allele diversity is faster than that in heterozygosity, which leads to an excess of heterozygosity under Hardy-Weinberg equilibrium in comparison with the expectations of mutation-drift equilibrium^75^. Bayesian simulations were carried out using three mutation models for microsatellites, infinite allele models (IAM), stepwise mutation model (SMM), and two-phase mutation model (TPM), with 30% multi-step mutations to avoid biases due to wrong assumptions about mutation models. We ran 1 000 000 MCMC and used the Wilcoxon sign-rank test to analyse the significance of differences in heterozygosities.

To test for models of historical gene flow among populations, we used a coalescent model implemented in MIGRATE-N 3.6.11^13^. Mutation-scaled effective population size (Θ = 4Ν_E_μ) and migration rates (M = m/μ), where μ is the mutation rate, were estimated using the Bayesian inference method. We considered μ = 4.76 × 10^−3^ mutations per locus per generation for the microsatellite loci^76^. The Brownian motion model for microsatellite data was defined as the mutation process of the markers and the prior distribution was set as uniform with Θ (10^−5^ to 100) and M (0 to 1 000). We ran 3 iterations with 2 000 000 MCMC after a burn-in of 50 000 generations, with sampling every 100 generations using a static heating scheme with four chains, swamping at every 10 chains based on an exponential temperature scheme. The independent runs were summarized to estimate the parameter values. The following models were tested: panmictic model, full migration model, zero-migration, a model with CON and ESM as one population, and a model with SER and CON as one population. We obtained the effective numbers of immigrants (N_E_*m*) using the equation (2) M * Θ = 4N_E_*m*, with the number 4 representing a scalar due to ploidy or inheritance of diploid nuclear data. The historical effective population size was estimated through the expression (3) (Θ/4μ) = Ν_E_.

### Species distribution models

To assess the current potential distribution of *D. exaltata*, species distribution models (SDM) were performed using 19 bioclimatic variables available in the WorldClim database, with resolution of ~1 km^29^. To obtain a subset of uncorrelated explanatory variables, an exploratory factor analysis (EFA) was used in the psych 1.7.5 package implemented in the R environment^77,78^. The algorithms Maxent, Generalized Linear Models (GLM), Generalized Boosted Models (GBM), Artificial Neural Network (ANN), Classification tree analysis (CTA), Surface Range Envelopes (SRE), Flexible Discriminant Analysis (FDA), Multiple Adaptive Regression Splines (MARS), and Random Forest (RF) were implemented in the Biomod2 3.3-7 package in the R environment^79^. To evaluate model performance, we randomly subset points for training (75%) and points to test the predictive power of the models (25%). Then, we used the receiver–operating characteristic (ROC) curve and its area under curve (AUC) and the true skill statistic (TSS) to select the best models. Consensus maps were built based on five independent runs of each algorithm using the TSS as a criterion to discard low-predictive-power models (TSS > 0.7). To evaluate the impacts of future climate change on the distribution of the species, we projected its potential distribution in 2050 using the same set of variables for MIROC-ESM and BCC-CSM1-1 projections in an arc resolution of 30 s representing an intermediary and a maximum representative concentration pathway for this period, respectively.

### Geospatial conservation assessment

The geospatial conservation status of *D. exaltata* was evaluated using the extent of occurrence (EOO) and area of occupancy (AOO), as estimated using the Geospatial Conservation Assessment Tool software^30^ (GeoCAT). Using this framework, we evaluated the conservation status of *D. exaltata* based on the International Union for the Conservation of Nature (IUCN) criteria B (EOO and AOO) to identify the threat category of the species^4^. The estimation of EOO was based on the minimum convex hull that contains all sites of known occurrence^30^. The estimation of AOO was based on the sum of squared grids of known occurrence based on grids of 2.0 km^2^ as suggested by IUCN guidelines^4,30^. The same dataset used in the SDM was used for geospatial analysis.

## Supplementary information

Supporting information

## Data Availability

The datasets generated during and/or analysed during the current study will be available in the DRYAD repository.

## References

[1] Brook BW, Sodhi NS, Bradshaw CJA (2008). Synergies among extinction drivers under global change. Trends Ecol. Evol..

[2] Krupnick GA (2013). Conservation of Tropical Plant Biodiversity: What Have We Done, Where Are We Going?. Biotropica.

[3] Oliver TH, Morecroft MD (2014). Interactions between climate change and land use change on biodiversity: Attribution problems, risks, and opportunities. Wiley Interdiscip Rev Clim Change.

[4] IUCN. Guidelines for using the IUCN Red List Categories and Criteria. Version 11 (February 2014). *Prepared by the Standards and Petitions Working Group of the IUCN SSC Biodiversity Assessments SubCommittee in August 2008* **1**, 86 (2014).

[5] Aguilar R, Quesada M, Ashworth L, Herrerias-Diego Y, Lobo J (2008). Genetic consequences of habitat fragmentation in plant populations: Susceptible signals in plant traits and methodological approaches. Mol. Ecol..

[6] Frankham, R., Ballou, J. D. & Briscoe, D. A. *Introduction to Conservation Genetics*. (Cambridge University press, 2010).

[7] Honnay O, Jacquemyn H (2007). Susceptibility of common and rare plant species to the genetic consequences of habitat fragmentation. Conserv. Biol..

[8] Angeloni F, Ouborg NJ, Leimu R (2011). Meta-analysis on the association of population size and life history with inbreeding depression in plants. Biol. Conserv..

[9] Reed DH (2005). Relationship between population size and fitness. Conserv. Biol..

[10] Reed DH, Frankham R (2003). Correlation between fitness and genetic diversity. Conserv. Biol..

[11] Waples, R. S. In *Population Viability Analysis.* 147–150 (University Chicago press, 2002).

[12] Hartl, D. L. & Clark, A. G. *Principles of Population Genetics*. (Sinauer Associates, 2007).

[13] Beerli P, Palczewski M (2010). Unified framework to evaluate panmixia and migration direction among multiple sampling locations. Genetics.

[14] Cornuet JM (2008). Inferring population history with DIY ABC: A user-friendly approach to approximate Bayesian computation. Bioinformatics.

[15] Nielsen R, Wakeley J (2001). Distinguishing migration from isolation: A Markov chain Monte Carlo approach. Genetics.

[16] Aavik T, Talve T, Thetloff M, Uuemaa E, Oja T (2017). Genetic consequences of landscape change for rare endemic plants – A case study of *Rhinanthus osiliensis*. Biol. Conserv..

[17] Chung MY, López-Pujol J, Chung MG (2017). The role of the Baekdudaegan (Korean Peninsula) as a major glacial refugium for plant species: A priority for conservation. Biol. Conserv..

[18] Hens H, Pakanen VM, Jäkäläniemi A, Tuomi J, Kvist L (2017). Low population viability in small endangered orchid populations: Genetic variation, seedling recruitment and stochasticity. Biol. Conserv..

[19] Sampson JF (2015). Long-term ‘islands’ in the landscape: Low gene flow, effective population size and genetic divergence in the shrub *Hakea oldfieldii* (Proteaceae). Bot. J. Linn. Soc..

[20] Wiberg RAW, Scobie AR, A’Hara SW, Ennos RA, Cottrell JE (2016). The genetic consequences of long term habitat fragmentation on a self-incompatible clonal plant, *Linnaea borealis* L. Biol. Conserv..

[21] Silva, M. F. Da. Flora Neotropica Monograph - *Dimorphandra*. 44, (1986).

[22] Matheus MT, Rodrigues-Junior AG, Oliveira DMT, Garcia QS (2017). Seed longevity and physical dormancy break of two endemic species of *Dimorphandra* from Brazilian biodiversity hotspots. Seed Sci. Res..

[23] Myers N, Mittermeier RA, Mittermeier CG, da Fonseca GA, Kent J (2000). Biodiversity hotspots for conservation priorities. Nature.

[24] Ribeiro MC, Metzger JP, Martensen AC, Ponzoni FJ, Hirota MM (2009). The Brazilian Atlantic Forest: How much is left, and how is the remaining forest distributed? Implications for conservation. Biol. Conserv..

[25] Klink CA, Machado RB (2005). Conservation of the Brazilian Cerrado. Conserv. Biol..

[26] Fernandes FM, Rego JO (2014). *Dimorphandra wilsonii* Rizzini (Fabaceae): distribution, habitat and conservation status. Acta Bot. Brasilica.

[27] Martins, E. M. *et al*. *Plano de Ação Nacional Para a Conservação do Faveiro-de-wilson (Dimorphandra wilsonii Rizzini)*. (Instituto de Pesquisas Jardim Botânico do Rio de Janeiro, 2014).

[28] Van Oosterhout C, Hutchinson WF, Wills DPM, Shipley P (2004). MICRO-CHECKER: Software for identifying and correcting genotyping errors in microsatellite data. Mol. Ecol. Notes.

[29] Hijmans RJ, Cameron SE, Parra JL, Jones PG, Jarvis A (2005). Very high resolution interpolated climate surfaces for global land areas. Int. J. Climatol..

[30] Bachman S, Moat J, Hill AW (2011). de laTorre, J. & Scott, B. Supporting red list threat assessments with GeoCAT: Geospatial conservation assessment tool. ZooKeys.

[31] JBRJ. Brazilian Flora 2020 in construction. Rio de Janeiro Botanical Garden Available at, http://floradobrasil.jbrj.gov.br/ (2017).

[32] Lewis, G. P. *Legumes of Bahia*. Kew R. Bot. Gard. xvi, 369p.-illus., col. illus., maps. *ISBN* 947643052 (1987).

[33] Resende-Moreira, L. C. *et al*. Gene flow between vicariant tree species: insights into savanna-forest evolutionary relationships. *Tree Genet. Genomes* **13** (2017).

[34] Sujii PS (2017). Recovery of genetic diversity levels of a Neotropical tree in Atlantic Forest restoration plantations. Biol. Conserv..

[35] Neto OC (2014). Genetic and ecological outcomes of I*nga vera* subsp. *affinis* (Leguminosae) tree plantations in a fragmented tropical landscape. PLoS One.

[36] Gonela A (2013). Genetic diversity and mating system of *Copaifera langsdorffii* (Leguminosae/Caesalpinioideae). Genet. Mol. Res..

[37] Manoel RO (2012). Contemporary pollen flow, mating patterns and effective population size inferred from paternity analysis in a small fragmented population of the Neotropical tree *Copaifera langsdorffii* Desf. (Leguminosae-Caesalpinioideae). Conserv. Genet..

[38] Sebbenn AM (2011). Low levels of realized seed and pollen gene flow and strong spatial genetic structure in a small, isolated and fragmented population of the tropical tree *Copaifera langsdorffii* Desf. Heredity (Edinb)..

[39] Batista Leite FA, Brandão RL, de Oliveira Buzatti RS, de Lemos-Filho JP, Lovato MB (2014). Fine-scale genetic structure of the threatened rosewood *Dalbergia nigra* from the Atlantic Forest: Comparing saplings versus adults and small fragment versus continuous forest. Tree Genet. Genomes.

[40] Buzatti RS (2012). Fine-scale spatial genetic structure of *Dalbergia nigra* (Fabaceae), a threatened and endemic tree of the Brazilian Atlantic Forest. Genet. Mol. Biol..

[41] Resende LC, Ribeiro RA, Lovato MB (2011). Diversity and genetic connectivity among populations of a threatened tree (*Dalbergia nigra*) in a recently fragmented landscape of the Brazilian Atlantic Forest. Genetica.

[42] Franceschinelli EV, Jacobi CM, Drummond MG, Resende MFS (2006). The genetic diversity of two Brazilian *Vellozia* (Velloziaceae) with different patterns of spatial distribution and pollination biology. Ann. Bot..

[43] Silva RM, da, Fernandes GW, Lovato MB (2007). Genetic variation in two *Chamaecrista* species (Leguminosae), one endangered and narrowly distributed and another widespread in the Serra do Espinhaço, Brazil. Can. J. Bot..

[44] Souza HAVe, Collevatti RG, Lima-Ribeiro MS, Lemos-Filho JPde, Lovato MB (2017). A large historical refugium explains spatial patterns of genetic diversity in a Neotropical savanna tree species. Ann. Bot..

[45] Frankham R, Bradshaw CJA, Brook BW (2014). Genetics in conservation management: Revised recommendations for the 50/500 rules, Red List criteria and population viability analyses. Biol. Conserv..

[46] ESA. ESA/CCI viewer. Available at, http://maps.elie.ucl.ac.be/CCI/viewer/download.php. (Accessed: 8th January 2018).

[47] Lowe WH, Allendorf FW (2010). What can genetics tell us about population connectivity?. Mol. Ecol..

[48] Vinson CC, Dal’Sasso TCS, Sudré CP, Mangaravite E, de Oliveira LO (2015). Population genetics of the naturally rare tree *Dimorphandra wilsonii* (Caesalpinioideae) of the Brazilian Cerrado. Tree Genet. Genomes.

[49] de Oliveira Buzatti RS, Lemos-Filho JP, Bueno ML, Lovato MB (2017). Multiple Pleistocene refugia in the Brazilian cerrado: Evidence from phylogeography and climatic nichemodelling of two *Qualea* species (Vochysiaceae). Bot. J. Linn. Soc..

[50] Pearson RG, Dawson TP (2003). Predicting the impacts of climate change on the distribution of species: Are bioclimate envelope models useful?. Glob. Ecol. Biogeogr..

[51] Novaes, R. M. L., Ribeiro, R. A., Lemos-Filho, J. P. & Lovato, M. B. Concordance between phylogeographical and biogeographical patterns in the Brazilian Cerrado: Diversification of the endemic tree *Dalbergia miscolobium* (Fabaceae). *PLoS One* **8** (2013). 10.1371/journal.pone.0082198 PMC3846898 24312640

[52] Ribeiro PCC, Lemos-Filho JPD, de Oliveira Buzatti RS, Lovato MB, Heuertz M (2016). Species-specific phylogeographical patterns and Pleistocene east-west divergence in *Annona* (Annonaceae) in the Brazilian Cerrado. Bot. J. Linn. Soc..

[53] Ribeiro PCC (2016). Climatic drivers of leaf traits and genetic divergence in the tree *Annona crassiflora*: a broad spatial survey in the Brazilian savannas. Glob. Chang. Biol..

[54] Aitken SN, Yeaman S, Holliday JA, Wang T, Curtis-McLane S (2008). Adaptation, migration or extirpation: climate change outcomes for tree populations. Evol. Appl..

[55] Frankham R (2015). Genetic rescue of small inbred populations: meta-analysis reveals large and consistent benefits of gene flow. Mol. Ecol..

[56] Http://www.splink.org.br. speciesLink: Sistema de Informação Distribuído para Coleções Biológicas. Available at, http://splink.cria.org.br/index?criaLANG=pt. (Accessed: 2nd January 2018).

[57] Pritchard JK, Stephens M, Donnelly P (2000). Inference of population structure using multilocus genotype data. Genetics.

[58] Hubisz MJ, Falush D, Stephens M, Pritchard JK (2009). Inferring weak population structure with the assistance of sample group information. Mol. Ecol. Resour..

[59] Doyle JJ, Doyle JL (1987). A rapid DNA isolation procedure for small quantities of fresh leaf tissue. Phytochem. Bull..

[60] Ferreira, M. A. & Grattapaglia, D. *Introdução ao uso de marcadores moleculares em análise genética* Embrapa. (1995).

[61] Souza, H. A. V, Collevatti, R. G., Lemos-Filho, J. P., Santos, F. R. & Lovato, M. B. Development of microsatellite markers for *Dimorphandra mollis* (Leguminosae), a widespread tree from the Brazilian cerrado. *Am. J. Bot*. 99 (2012). 10.3732/ajb.1100413 22343542

[62] Vinson C, Azevedo V, Mendonça M, Ciampi A, Oliveira O (2013). Microsatellite markers for the rare tree *Dimorphandra wilsonii* (Caesalpinioideae, Fabaceae) and transferability to *Dimorphandra* species. Mol. Ecol. Resour..

[63] Goudet, J. FSTAT (version 2.9. 3.2): a program to Estimate and Test gene Diversities and Fixation Indices. http://www.unil.ch/izea/software/fstat.html (2002).

[64] Excoffier L, Lischer HEL (2010). Arlequin suite ver 3.5: A new series of programs to perform population genetics analyses under Linux and Windows. Mol. Ecol. Resour..

[65] Mousadik AE, Petit RJ (1996). High level of genetic differentiation for allelic richness among populations of the argan tree [*Argania spinosa* (L.) Skeels] endemic to Morocco Title. Theor. Appl. Genet..

[66] Peakall, R., Smouse, P. E., Rod, P. & Peter, E, S. GenAlEx 6: Genetic analysis in Excel. Population genetic software for teaching and research. *Mol. Ecol. Notes*, 10.1111/J.1471-8286.2005.01155.X (2006).

[67] Peakall, R. & Smouse, P. E. Genalex 6: genetic analysis in Excel. *Mol. Ecol. Notes* (2006). 10.1093/bioinformatics/bts460 PMC3463245 22820204

[68] Evanno G, Regnaut S, Goudet J (2005). Detecting the number of clusters of individuals using the software STRUCTURE: A simulation study. Mol. Ecol..

[69] Jakobsson M, Rosenberg NA (2007). CLUMPP: A cluster matching and permutation program for dealing with label switching and multimodality in analysis of population structure. Bioinformatics.

[70] Rosenberg NA (2004). DISTRUCT: A program for the graphical display of population structure. Mol. Ecol. Notes.

[71] Cockerham, C. C. Variance of Gene Frequencies. *Evolution* (N. Y). **23**, 72 (1969). 10.1111/j.1558-5646.1969.tb03496.x 28562963

[72] Lindgren D, Mullin TJ (1998). Relatedness and status number in seed orchard crops. Can. J. For. Res..

[73] Hardy OJ, Vekemans X (2002). SPAGeDi: a versatile computer program to analyse spatial genetic structure at the individual or population levels. Mol. Ecol. Notes.

[74] Piry S, Luikart G, Cornuet JM (1999). BOTTLENECK: A computer program for detecting recent reductions in the effective population size using allele frequency data. J. Hered..

[75] Cornuet JM, Luikart G (1996). Description and power analysis of two tests for detecting recent population bottlenecks from allele frequency data. Genetics.

[76] Cieslarová J, Hanáček P, Fialová E, Hýbl M, Smýkal P (2011). Estimation of pea (Pisum sativum L.) microsatellite mutation rate based on pedigree and single-seed descent analyses. J. Appl. Genet..

[77] R Core Team. R Development Core Team. R: A Language and Environment for Statistical Computing **55**, 275–286 (2016).

[78] Revelle, W. Psych: Procedures for Personality and Psychological Research. *R Packag*. 1–358 (2016).

[79] Thuiller, W., Georges, D. & Engler, R. Biomod2: Ensemble platform for species distribution modeling. *R Packag*. version 2, r560 (2013).

