# Supplementary material for: Genetic data improve the assessment of the conservation status based only on herbarium records of a Neotropical tree
Source: Sci Rep. 2019 Apr 5;9:5693. doi: 10.1038/s41598-019-41454-0 (PMC6451013; doi:10.1038/s41598-019-41454-0)
Supplement: Supplementary file 1 — Supporting information [file 41598_2019_41454_MOESM1_ESM.docx]

ORIGINAL ARTICLE

**Title: Genetic data improve the assessment of the conservation status based only on herbarium records of a neotropical tree**

André Carneiro Muniz1, José Pires Lemos-Filho2, Renata Santiago de Oliveira Buzatti1, Priciane Cristina Correa Ribeiro1,4, Fernando Moreira Fernandes3, Maria Bernadete Lovato1,*

1Departamento de Biologia Geral, Universidade Federal de Minas Gerais, CP 486, Belo Horizonte, MG 31270-901, Brazil

2Departamento de Botânica, Universidade Federal de Minas Gerais, Belo Horizonte, MG 31270-901, Brazil

3Jardim Botânico da Fundação Municipal de Parques e Zoobotânica de Belo Horizonte, Belo Horizonte, Brazil; Coordination of Plano de Ação Nacional para a Conservação do faveiro-de-wilson

4Present address: Universidade Federal do Tocantins, Campus Universitário Araguaína. Araguaína, TO 77824-838, Brazil

*Corresponding author

E-mail address: lovatomb@icb.ufmg.br; Telephone number: + 55 (31) 34092571; postal address: Departamento de Biologia Geral, Universidade Federal de Minas Gerais, CP 486, Belo Horizonte, MG, 31270-901, Brazil

**Supporting information**

Supplementary table 1: Pairwise genetic divergence based on *F_ST_* (inferior diagonal) and *R_ST_* (superior diagonal) between *Dimorphandra exaltata* populations. Significant values (P < 0.05) are in bold.

|  | SER | CON | ESM | AFS |
| --- | --- | --- | --- | --- |
| SER | 0.000 | 0.006 | **0.059** | **0.243** |
| CON | **0.098** | 0.000 | **0.067** | **0.322** |
| ESM | **0.184** | **0.155** | 0.000 | **0.351** |
| AFI | **0.239** | **0.274** | **0.294** | 0.000 |

Supplementary table 2: Model evaluation of coalescent analysis for *Dimorphandra exaltata* performed in Migrate-n program. LBF = Bayes factor.

| Model | Log likelihood | LBF | Model-probability |
| --- | --- | --- | --- |
| Full model | -541.69 | 1.000 | 1.000 |
| Model CON + ESM | -750.1 | 3.08E-91 | 3.25E+90 |
| Model SER + ESM | -730.77 | 7.65E-83 | 1.31E+82 |
| Panmitic Model | -987.91 | 1.62E-194 | 6.18E+193 |
| No migration | -936.71 | 9.04E-82 | 1.11E+81 |

Supplementary table 3: Demographic parameters of *Dimorphandra exaltata* populations estimated by coalescent analysis in Migrate-n for the full migration matrix model. N_E_ = historical effective population size, N_E_*m* = effective number of migrants arriving into the populations of the first column. Values within the brackets are the 95% confidence intervals of the posterior distribution.

|  | N_E_ |  | N_E_*m* |  |
| --- | --- | --- | --- | --- |
| Pop |  | SER | CON | ESM |
| SER | 481 (0-1384) | - | 3.514(0.00-22.20) | 5.653(0.00-25.8) |
| CON | 760 (96-1376) | 1.925(0.00-20.90) | - | 2.975(0.00-21.53) |
| ESM | 11 (0-724) | 0.086(0.00-13.33) | 0.108(0.0-15.3) | - |

Supplementary table 4: Species distribution model evaluation for *Dimorphandra exaltata* based on True Skill Statistics for each run of algorithms tested. Generalized Linear Models (GLM), Generalized Boosted Models (GBM), Artificial Neural Network (ANN), Classification tree analysis (CTA), Surface Range Envelopes (SRE), Flexible Discriminant Analysis (FDA), Multiple Adaptive Regression Splines (MARS) and Random Forest (RF).

| Algorithms | RUN1 | RUN2 | RUN3 | RUN4 | RUN5 | Full |
| --- | --- | --- | --- | --- | --- | --- |
| GLM | 0.944 | 0.611 | 1.000 | 0.667 | 0.778 | 0.788 |
| GBM | 0.889 | 0.500 | 0.861 | 0.500 | 0.806 | 0.983 |
| SER | 0.861 | 0.556 | 0.861 | 0.500 | 0.333 | 0.696 |
| ANN | 0.389 | 0.556 | 0.861 | 0.333 | 0.889 | 0.356 |
| CTA | 0.528 | 0.333 | 0.583 | 0.556 | 0.500 | 0.772 |
| RF | 0.917 | 0.500 | 0.944 | 0.444 | 0.667 | 0.978 |
| MARS | 0.917 | 0.556 | 0.972 | 0.639 | 0.944 | 0.967 |
| FDA | 0.889 | 0.667 | 0.917 | 0.861 | 0.528 | 0.739 |
| MAXENT | 0.944 | 0.639 | 0.972 | 0.667 | 0.722 | 0.805 |

Supplementary table 5: Species distribution model evaluation for *Dimorphandra exaltata* based on area under curve of receiving operate characteristic for each run of algorithms tested. Generalized Linear Models (GLM), Generalized Boosted Models (GBM), Artificial Neural Network (ANN), Classification tree analysis (CTA), Surface Range Envelopes (SRE), Flexible Discriminant Analysis (FDA), Multiple Adaptive Regression Splines (MARS) and Random Forest (RF).

| Algorithms | RUN1 | RUN2 | RUN3 | RUN4 | RUN5 | Full |
| --- | --- | --- | --- | --- | --- | --- |
| GLM | 0.981 | 0.731 | 1.000 | 0.801 | 0.870 | 0.942 |
| GBM | 0.944 | 0.620 | 0.954 | 0.722 | 0.889 | 0.995 |
| SER | 0.931 | 0.778 | 0.931 | 0.750 | 0.667 | 0.848 |
| ANN | 0.667 | 0.611 | 0.898 | 0.620 | 0.963 | 0.628 |
| CTA | 0.759 | 0.667 | 0.806 | 0.856 | 0.727 | 0.903 |
| RF | 0.940 | 0.620 | 0.981 | 0.690 | 0.861 | 0.999 |
| MARS | 0.954 | 0.722 | 0.972 | 0.782 | 0.954 | 0.975 |
| FDA | 0.972 | 0.755 | 0.981 | 0.935 | 0.685 | 0.915 |
| MAXENT | 0.981 | 0.787 | 0.991 | 0.815 | 0.806 | 0.944 |

Supplementary table 6: Mean variable contribution to species distribution models for *Dimorphandra exaltata* for each algorithm. Bio2 = mean diurnal range temperature, Bio3 = isothermality, Bio10 = mean temperature of warmest quarter, Bio16 = precipitation of wettest quarter, Bio17 = precipitation of driest quarter.

| Variable | GLM | GBM | SRE | ANN | CTA | RF | MARS | FDA | MAXENT |
| --- | --- | --- | --- | --- | --- | --- | --- | --- | --- |
| Bio2 | 0.052 | 0.036 | 0.202 | 0.604 | 0.066 | 0.089 | 0.348 | 0.000 | 0.004 |
| Bio3 | 0.568 | 0.323 | 0.464 | 0.474 | 0.532 | 0.303 | 0.674 | 0.609 | 0.834 |
| Bio10 | 0.429 | 0.390 | 0.402 | 0.635 | 0.348 | 0.401 | 0.176 | 0.407 | 0.103 |
| Bio16 | 0.000 | 0.095 | 0.337 | 0.564 | 0.301 | 0.145 | 0.139 | 0.000 | 0.004 |
| Bio17 | 0.520 | 0.248 | 0.410 | 0.671 | 0.206 | 0.152 | 0.573 | 0.360 | 0.486 |

| Supplementary table 7: Herbarium records of *Dimorphandra exaltata* used in the species distribution modeling and in the Geospatial Conservation Assessment Tool. | | | |  | |  |  |
| --- | --- | --- | --- | --- | --- | --- | --- |
| County/State | Longitude | Latitude | Voucher Number | |  |  |  |
| Jequié/BA | 41 03' W | 13 56' S | HUESB-1895 | |  |  |  |
| Caratinga/MG | 42 08' W | 19 47' S | BHCB-13465 | |  |  |  |
| Carmo do Cajuru/MG | 44 42' W | 20 09' S | BHZB-8162 | |  |  |  |
| Contagem/MG | 44 04' W | 19 51' S | BHCB-174517 | |  |  |  |
| Florestal/MG | 44 29' W | 19 54' S | BHZB7787 | |  |  |  |
| Florestal/MG | 44 24' W | 19 54' S | BHZB-8418 | |  |  |  |
| Fortuna de Minas/MG | 44 26' W | 19 33' S | BHZB-7098 | |  |  |  |
| Lagoa da Prata/MG | 45 01' W | 19 03' S | BHZB-10274 | |  |  |  |
| Matozinhos/MG | 44 06' W | 19 35' S | BHZB-7773 | |  |  |  |
| Piau/MG | 43 19' W | 21 30' S | UB-127842 | |  |  |  |
| Ribeirão das Neves/MG | 44 05' W | 19 46' S | BHZB-4396 | |  |  |  |
| Sabará/MG | 43 47' W | 19 49' S | BHZB-8466 | |  |  |  |
| Serro/MG | 43 22' W | 18 36' S | BHZB-8082 | |  |  |  |
| Viçosa/MG | 42 52' W | 20 45' S | VIC-40541 | |  |  |  |
| Rio de Janeiro/RJ | 43 12' W | 22 54' S | NYBG_BR-777859 | |  |  |  |
| Valença/RJ | 43 42' W | 22 14' S | RBR-5033 | |  |  |  |
| Campinas/SP | 47 03' W | 22 54' S | IAC 19699 | |  |  |  |
| Piracicaba/SP | 47 38' W | 22 43' S | SP-97467 | |  |  |  |
| Rio Claro/SP | 47 33' W | 22 24' S | HRCB-15249 | |  |  |  |


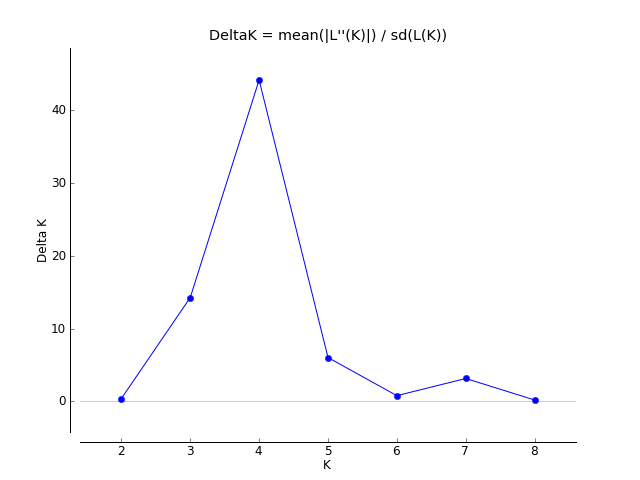

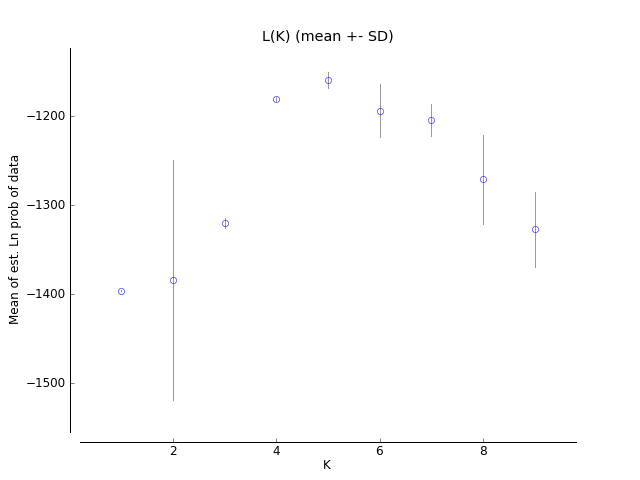


B

A

Supplementary figure 1. Evaluation of the best number of K based on the results of Bayesian clustering method for the three *Dimophandra exaltata* populations and seven isolated individuals with (A) delta K method and (B) mean Log Likelihood of successive K.


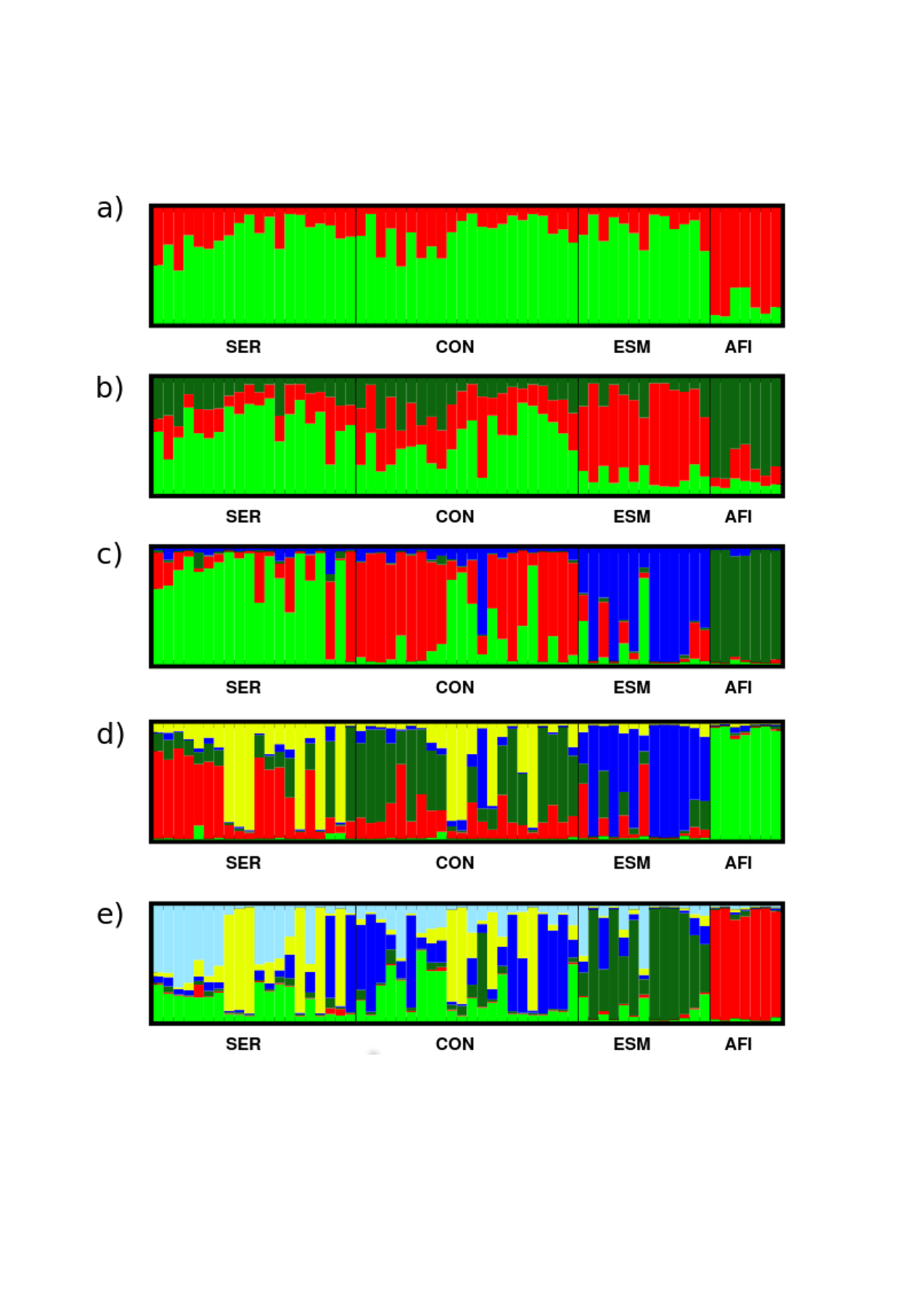


Supplementary figure 2. Barplots for individuals membership coefficients for sequential K values based on the Bayesian clustering method implemented for STRUCTURE software for *Dimorphandra exaltata* populations. a) K=2, b) K=3, c) K=4, d) K=5, e) K=6.
